# Postgraduate education in dental implantology in the United Kingdom: a review

**DOI:** 10.1186/s40729-017-0115-1

**Published:** 2018-01-29

**Authors:** Na Yeoun Kim, Sami Stagnell

**Affiliations:** 1 Dover Health Centre Dental Department, Maidson Dieu Road, Dover, CT16 1RH UK; 2 Southampton NHS Treatment Centre, Royal South Hants Hospital, Brinton Terrace, Southampton, Hampshire SO14 0YG UK

**Keywords:** Implantology, Postgraduate education, United Kingdom, Educational standards

## Abstract

**Background:**

The qualified dentists in the United Kingdom (UK) are not expected to be competent in practising implant dentistry without further training in the subject and there is now greater emphasis on postgraduate training in Dental Implantology. There are three main education pathways at present, yet their training standards vary significantly.

This study aims to identify UK postgraduate academic qualifications and continuing professional development (CPD) courses available in the field of Dental Implantology and evaluates the current standard of the postgraduate training programmes against the Training Standards in Implant Dentistry (TSID) guidelines from Faculty of General Dental Practice (FGDP (UK)).

**Results:**

There were eight master level courses with varying types of qualification and study mode. The mean duration and tuition fee of the courses were 2.50 years and £23,635.50 per course, respectively. There were eight postgraduate diploma part-time courses with the mean duration of 2.00 years, and the mean tuition fee of £20,177.08 per course. The mean duration for two postgraduate certificate part-time courses was 1.00 year with the mean tuition fee of £9441.50. However, there were no full-time study options for these courses. All courses identified stated their compliance with TSID guidelines.

The mean duration for 13 CPD courses identified was 0.94 years and all courses were delivered in a part-time mode. Eleven of these courses were verifiable CPD courses, and two courses were providing certificates only. Not all courses were fully compliant with TSID guidelines. Ten courses clearly stated that they provide mentoring for implant placements, and the number of supervised cases varied considerably between 1 and 50.

**Conclusion:**

Development of FGDP (UK) TSID guidelines has led to a significant improvement in the quality of postgraduate education in Dental Implantology in the UK. However, not all courses are fully compliant with these guidelines and the provision of mentoring for implant placements also needs to be standardised. Quality-assured training is directly related to patient safety, and therefore all UK postgraduate training pathways must ensure their compliance with the current guidelines.

## Review

### Background

The global dental implants and prosthetics market is expected to reach USD 12.32 billion by 2021, at a compound annual growth rate of 7.2% from 2016 to 2021 [1]. Although Europe accounted for the largest share of the global dental implants and prosthetics market in 2015 [1], the number of implants placed per 10,000 people in the United Kingdom (UK) is significantly lower than many comparable European nations [2]. It is believed that the UK is one of the European countries with the greatest potential for an increase in the use of dental implants [2], and this has been reflected by the 20–30% rapid growth of dental implant industry in the UK within the last few years [3].

However, this also accompanied an increase in the number of litigation cases arising from implant dentistry. According to the statistics provided by one of the largest indemnity organisations in the UK, ‘implants’ claims and ‘implants and perio’ claims accounted for 28.8 and 5.5%, respectively, based on the top 20 UK claims by value [4].

The ‘Scope of Practice’ from the General Dental Council (GDC) identifies providing dental implants as an additional skill that a dentist could develop [5]. The qualified general dental practitioners in the UK are not expected to be competent in practicing implant dentistry without further training in the subject, and the GDC emphasises the importance of appropriate postgraduate training prior to practicing dental implants. Dental Implantology is not recognised as a specialty in the UK [6], and the current education pathways for the subject include:

1. Specialist training in a related specialty of dentistry such as periodontology and prosthodontics;
2. Academic qualification at a postgraduate level;
3. Continuing professional development (CPD) courses [7].

Development of Training Standards in Implant Dentistry (TSID) guidelines by Faculty of General Dental Practice (FGDP (UK)) [8] has led to a significant improvement in the quality of postgraduate education in Dental Implantology in the UK. The latest review in 2016 states that it aims to provide a summary of the training that a reasonable dental practitioner carrying out safe implant dentistry in the UK should undertake, before embarking upon patient care in this discipline. This document is not only used by the education providers and prospective students for the postgraduate training purposes but also by the GDC when investigating the fitness to practice of dental practitioners, who have allegedly practised implant dentistry beyond the limits of their own competence.

#### Aim

This article aims to evaluate the current standards of the postgraduate education in Dental Implantology in the UK by identifying all postgraduate academic qualifications and CPD courses available in the field of Dental Implantology and comparing their core modules against the FGDP (UK) TSID 2016 guidelines.

### Method

Completion of specialist training in another field of dentistry, such as oral surgery, periodontology and prosthodontics, is a training pathway for Dental Implantology and would certainly prepare graduate dentists as competent practitioners. However, the authors felt that this pathway does not fit the purpose of this study, and hence, only the academic courses and CPD courses in Dental Implantology were considered.

#### Stage 1: Identification of courses in Dental Implantology

##### Academic courses

Initial search for postgraduate academic courses related to Dental Implantology was carried out via Universities and Colleges Admissions Services (UCAS) website in January 2017. During the initial search, it was evident that there was a variation in the titles being used to describe the postgraduate training in Dental Implantology, and hence, several different terms were used to identify all relevant courses. The terms of search included in this study are:

- Implantology
- Dental Implantology
- Implant Dentistry
- Clinical Implant Dentistry
- Clinical Dentistry (Implantology).

In addition to the courses that were identifiable on the UCAS website, those that were identified through online search engines and professional dental journals were also included in the study.

##### CPD courses

The CPD courses in Dental Implantology were identified in a similar manner. The primary search was carried out via online search engines in January 2017, and those that were identified from professional dental journals were also included in the study. Any short CPD courses that did not have any practical component or lacking implant placement experience have been excluded from the analysis for this reason.

#### Stage 2: Review of the core modules of identified courses

The core modules of both the identified academic courses CPD courses were compared against the standards specified in TSID 2016 guideline. The main standards from the guideline are listed in Table 1.

**Table 1:** Training standards in FGDP (UK) TSID 2016 guideline

Training Standards in Implant Dentistry, 2016	
1	Basic sciences: surgical anatomy, pathological process, bone defects, healing processes
2	Implant science: design and materials, limitations
3	Patient assessment and medical considerations
4	Case assessment and treatment planning: straightforward and complex cases
5	Radiographic assessment and Ionising Radiation Medical Exposure Regulations (IRMER)
6	Communication and informed consent
7	Infection control and asepsis
8	Soft tissue management (raising flaps and suturing)
9	Hard tissue management (autogenous bone augmentation, guided bone regeneration)
10	Pharmacological management
11	Surgical techniques for implant placement procedures
12	Implant-supported restorative procedures
13	Complications and their management
14	Long-term monitoring of implants
15	Record-keeping, documentation and quality assurance

### Results

#### Academic courses

All the academic courses identified from this process were included in this study, and a total of 18 courses were included in the study. The list of ten dental institutions offering postgraduate academic courses in Dental Implantology is shown in Table 2.

**Table 2:** List of dental institutions offering postgraduate academic courses in Dental Implantology

Dental institutions
BPP University
Cardiff University
Edge Hill University
Faculty of General Dental Practitioners
Newcastle University
The City of London Dental School
University of Bristol
University of Central Lancashire
University of Manchester
University of Sheffield

There were eight master level courses with three full-time options and five part-time options, and the mean duration of the courses was 2.50 years. The type of the qualification varied between Master of Science (MSc), Master of Clinical Dentistry (MClinDent), and Master of Medical Science (MMedSci). The mean tuition fee was £23,635.50 per course. This figure refers to the tuition fee itself only and does not include any additional bench fees; the actual cost that incurs until completion of the course may therefore be greater than the figures provided.

In addition, postgraduate diploma and postgraduate certificate qualifications were also available in Dental Implantology. There were eight postgraduate diploma part-time courses with the mean duration of 2.00 years, and the mean tuition fee was £20,177.43 per course. The mean duration for two postgraduate certificate part-time courses was 1.00 year with the mean tuition fee of £9441.50. However, there were no full-time study options for these courses (Table 3).

**Table 3:** Summary of findings from analysis of academic courses identified

Level of qualification	Mean duration (years)	Study mode		Mean tuition fee—per course (£)
		FT	PT	
Masters level	2.50	3	5	23,635.50
Postgraduate diploma	2.00	N/A	8	20,177.43
Postgraduate certificate	1.00	N/A	2	9441.50

The entry requirement for all postgraduate academic courses identified was also explored. All institutions required a primary qualification in dentistry and full registration with GDC. However, the minimum number of years of post-qualification clinical experience varied between institutions; six out of ten institutions required a minimum of 2 years post-qualification clinical experience, two institutions required 1-year experience, one institution did not require any, and one institution did not state whether they require any post-qualification clinical experience.

Detailed review of the core modules of identified academic courses was impossible based on the information that was available online. However, all courses stated that they were compliant with the FGDP (UK) TSID 2016 guideline and GDC requirements.

#### CPD courses

A total of 13 CPD courses were included in this study. The mean duration for CPD courses was 0.94 years, and all courses were delivered in part-time mode. The mean tuition fee per course was £7543.08. Eleven of these courses were verifiable CPD courses, and two courses were providing certificates only, which would be considered as non-verifiable CPD courses. Of 11 institutions who declared that they provide verifiable CPD courses, 10 institutions stated the exact number of CPD hours delivered throughout their courses; the average number of CPD hours delivered amongst these institutions was 81.00 h (Table 4).

**Table 4:** Summary of findings from analysis of CPD courses identified

Qualification	Duration (years)	Study mode	Mean tuition fee—per course (£)
CPD (11 verifiable, 2 non-verifiable)	0.94	13 PT	7543.08
Average CPD hours: 81.00 (*n* = 10)			

There were four courses that stated the number of intake in each cohort, and this varied between 4 and 24. Ten courses also provided the profiles of the trainers or mentors on their website. Ten courses clearly stated that they are able to provide mentoring for implant placements on patients; seven courses had mentoring component throughout the course and three offered it following completion of the course. Interestingly, the number of patients supervised varied significantly between 1 and 50. Of the remaining three courses, two courses did not offer any mentoring opportunities and one did not clarify this on their website.

Most of CPD courses identified had a modular learning structure of theoretical components of Dental Implantology. They typically consisted of 6 to12 study days on a monthly basis, and these sessions also covered practical components such as placement of implants on plastic jaw models. The clinical mentoring was offered in various different settings depending on the arrangement made by the provider. This included regional training centres as well as the trainee’s own practice where they place an implant under supervision of accredited mentors visiting their practices.

**Fig. 1:**
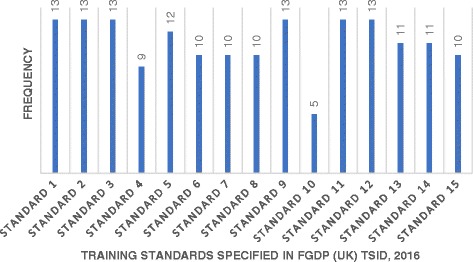
The frequency of each training standard covered by CPD courses

The standards from FGDP (UK) TSID 2016 guideline covered by the identified CPD courses are summarised in Fig. 1.

### Discussion

#### Limitations of the study

Due to the design of the study, the data collection was only based on the information available online or in dental journals. This inevitably carried the risk of the information being outdated or exclusion of further details that were available via other methods. This was particularly relevant when reviewing the core modules of the academic courses identified. A considerable number of courses only displayed the title of the core modules without further expansion on topics covered within each module. Hence, detailed review of the core modules and direct comparison of identified courses were impossible. This explains why the findings had to rely on statements from the institutions confirming whether or not the courses were compliant with the FGDP (UK) TSID 2016 guideline and GDC requirements.

#### Provision of dental implants in the UK

‘Guidelines for selecting appropriate patients to receive treatment with dental implants: priorities for the NHS [9]’ is a national guideline provided by the Royal College of Surgeons of England (RCS Eng) that specifies the selection criteria for National Health Service (NHS) dental implant therapy in the UK. This document details eight main groups of patients who should be prioritised for treatment with dental implants within the NHS (Table 5).

**Table 5:** Selection criteria for NHS dental implant therapy

Selection criteria for NHS dental implant therapy	
1	People with congenital conditions resulting in deformed and/or missing teeth
2	People who have lost teeth due to trauma
3	People who have undergone ablative surgery for head and neck cancer
4	People with extraoral defects
5	People who are edentulous in one or both jaws
6	People with severe denture intolerance
7	People with aggressive periodontitis
8	People with malocclusions requiring implant-borne anchorage

The demand for dental implants from patients outside these priority groups had to be met by the private sector and this subsequently led to an increase in the number of dental practitioners practising Dental Implantology in the recent years. According to Ucer in his article ‘Educational Pathways in Implant Dentistry in the UK’ [7], an unpublished research had shown that most dental practitioners place 20–50 implants per annum, while a small number of referral dentists place 200 to 400 yearly.

The number of specialists in periodontology and prosthodontics were 377 and 446, respectively, according to the statistics provided by the GDC. However, these figures do not include specialists in other specialties who may be practising implant dentistry, which refers to 294 restorative specialists and 734 specialist oral surgeons. Moreover, these figures are immensely underestimated in predicting the number of dental practitioners practising Dental Implantology because they do not account for those who are not on the GDC specialist register and the appropriately trained practitioners who practise Dental Implantology following completion of academic or CPD courses.

#### Need for standardisation in postgraduate education

This study revealed the variation that exists amongst the postgraduate academic and CPD courses in Dental Implantology in terms of level of qualification, duration, study mode, tuition fee, entry requirements and core modules. The level of qualification ranged from non-verifiable CPD to masters level, and the duration of courses also varied in accordance with the level of qualifications. Most of the courses were delivered in a part-time mode which demonstrates the demand for such courses by the dental practitioners who are in full-time employment. The variation in tuition fees ranged drastically between programmes with some up to four times more expensive in both academic and CPD courses.

With respect to entry requirements, most institutions required the candidates to have up to 2 years post-qualification clinical experience (with no specification as to what experience was required), whereas the others only specified to have a primary qualification in Dentistry and registration with GDC. Some also included arrangement of professional indemnity as a part of entry requirements.

In addition, the availability of mentoring for implant placements on patients is another important factor within postgraduate education. In this study, the number of patients supervised during a CPD varied significantly from 1 patient to 50 patients. Furthermore, there were two courses that did not offer any mentoring opportunities at all. It is felt that the postgraduate courses without provision of mentoring or clinical supervision have limited benefit, and therefore, there is a need for more consistent approach to postgraduate training in the subject.

#### Regulation and quality assurance of postgraduate education

The dentists who practise Dental Implantology may possess different qualifications and different level of experiences depending on the way they developed their career pathway. Although all practitioners should aim to obtain a formal postgraduate qualification in the subject, it has been reported that the product training organised by commercial companies, short 1 to 2 day courses (with no predetermined learning outcomes) and study club meetings were ranked as the three common forms of CPD education in implant dentistry in Europe [10]. Practitioners should ensure to gain appropriate level of training and work within their competencies, as inadequate training and lack of skills may endanger patient safety.

Teaching experience and formal qualifications of speakers were shown to be the most important criterion for quality assurance of the courses, and it is fundamental that more nationally or internationally accredited courses with quality teaching staff become widely available throughout the country. In addition, all courses should have clear aims and learning objectives as well as formal end-of-course assessments, and participant feedback should be obtained regularly to monitor the quality of training.

Although there is no agreed definition of a mentor and their role at present, it is the prospective learner’s responsibility to check the background and calibration of the educating staff. They must ensure that the programme is well-structured with adequate amount of quality-assured mentoring available throughout the course.

## Conclusion

Development of FGDP (UK) TSID guidelines has led to a significant improvement in the quality of postgraduate education in Dental Implantology in the UK. However, not all courses are fully compliant with these guidelines, and the provision of mentoring for implant placements also needs to be standardised. Quality-assured training is directly related to patient safety, and therefore, all UK postgraduate training pathways must ensure their compliance with the current guidelines.

## Abbreviations

CPD: Continuing professional development
FGDP (UK): Faculty of General Dental Practice (United Kingdom)
GDC: General Dental Council
NHS: National Health Service
RCS Eng: Royal College of Surgeons of England
TSID: Training Standards in Implant Dentistry
UCAS: Universities and Colleges Admissions Services
UK: United Kingdom
